# Risk factors for mortality in patients admitted to intensive care units with pneumonia

**DOI:** 10.1186/s12931-016-0397-5

**Published:** 2016-07-11

**Authors:** Guowei Li, Deborah J. Cook, Lehana Thabane, Jan O. Friedrich, Tim M. Crozier, John Muscedere, John Granton, Sangeeta Mehta, Steven C. Reynolds, Renato D. Lopes, Lauzier Francois, Andreas P. Freitag, Mitchell A. H. Levine, the PROTECT Investigators for the Canadian Critical Care Trials Group, and the Australian and New Zealand Intensive Care Society Clinical Trials Group

**Affiliations:** Department of Clinical Epidemiology & Biostatistics, McMaster University, 501-25 Charlton Avenue East, Hamilton, ON L8N 1Y2 Canada; St. Joseph’s Healthcare Hamilton, McMaster University, 501-25 Charlton Avenue East, Hamilton, ON L8N 1Y2 Canada; Department of Medicine, McMaster University, Hamilton, ON Canada; Interdepartmental Division of Critical Care, Hamilton Health Sciences, Hamilton, ON Canada; Interdepartmental Division of Critical Care Medicine, University of Toronto, Toronto, ON Canada; St. Michael’s Hospital, University of Toronto, Toronto, ON Canada; Intensive Care Unit, Monash Medical Centre, Melbourne, VIC Australia; Department of Critical Care Medicine, Queens University Kingston, Kingston, ON Canada; University Health Network, University of Toronto, Toronto, ON Canada; Mount Sinai Hospital, University of Toronto, Toronto, ON Canada; Division of Critical Care, Department of Medicine, University of British Columbia, Vancouver, BC Canada; Duke Clinical Research Institute, Duke University, Durham, NC USA; Department of Anesthesiology and Critical Care Medicine, Division of Critical Care Medicine, Université Laval, Québec, Canada; Department of Clinical Epidemiology & Biostatistics, McMaster University, 25 Main St. West, Suite 2000, 20th floor, Hamilton, ON L8P 1H1 Canada; Centre for Evaluation of Medicines, St. Joseph’s Healthcare Hamilton, 25 Main St. West, Suite 2000, 20th floor, Hamilton, ON L8P 1H1 Canada

**Keywords:** Pneumonia, Intensive care, Mortality, Risk factor

## Abstract

**Background:**

Despite the high mortality in patients with pneumonia admitted to an ICU, data on risk factors for death remain limited.

**Methods:**

In this secondary analysis of PROTECT (Prophylaxis for Thromboembolism in Critical Care Trial), we focused on the patients admitted to ICU with a primary diagnosis of pneumonia. The primary outcome for this study was 90-day hospital mortality and the secondary outcome was 90-day ICU mortality. Cox regression model was conducted to examine the relationship between baseline and time-dependent variables and hospital and ICU mortality.

**Results:**

Six hundred sixty seven patients admitted with pneumonia (43.8 % females) were included in our analysis, with a mean age of 60.7 years and mean APACHE II score of 21.3. During follow-up, 111 patients (16.6 %) died in ICU and in total, 149 (22.3 %) died in hospital. Multivariable analysis demonstrated significant independent risk factors for hospital mortality including male sex (hazard ratio (HR) = 1.5, 95 % confidence interval (CI): 1.1 - 2.2, *p*-value = 0.021), higher APACHE II score (HR = 1.2, 95 % CI: 1.1 - 1.4, *p*-value < 0.001 for per-5 point increase), chronic heart failure (HR = 2.9, 95 % CI: 1.6 - 5.4, *p*-value = 0.001), and dialysis (time-dependent effect: HR = 2.7, 95 % CI: 1.3 - 5.7, *p*-value = 0.008). Higher APACHE II score (HR = 1.2, 95 % CI: 1.1 - 1.4, *p*-value = 0.002 for per-5 point increase) and chronic heart failure (HR = 2.6, 95 % CI: 1.3 – 5.0, *p*-value = 0.004) were significantly related to risk of death in the ICU.

**Conclusion:**

In this study using data from a multicenter thromboprophylaxis trial, we found that male sex, higher APACHE II score on admission, chronic heart failure, and dialysis were independently associated with risk of hospital mortality in patients admitted to ICU with pneumonia. While high illness severity score, presence of a serious comorbidity (heart failure) and need for an advanced life support (dialysis) are not unexpected risk factors of mortality, male sex might necessitate further exploration. More studies are warranted to clarify the effect of these risk factors on survival in critically ill patients admitted to ICU with pneumonia.

**Trial registration:**

ClinicalTrials.gov Identifier: NCT00182143.

**Electronic supplementary material:**

The online version of this article (doi:10.1186/s12931-016-0397-5) contains supplementary material, which is available to authorized users.

## Background

Pneumonia is the leading cause of death from infectious diseases globally [1]. Despite advances in antibiotics and vaccines, as well as publication of guidelines for the management of patients with hospital-acquired and community-acquired pneumonia [2–4], the mortality rate for patients admitted to intensive care unit (ICU) with pneumonia remains substantial, ranging from approximately 15 to 50 % [5–8].

Many studies have focused on prevention of and risk factors for pneumonia among patients admitted to the hospital [9, 10] or ICU [6, 11]. Previous studies have identified advanced age, renal failure, septic shock and acute respiratory distress syndrome as significant risk factors for pneumonia [12–15]. However, among patients admitted to ICU due to pneumonia, data on risk factors for death remain limited. Furthermore, the effects of some proposed risk factors such as gender [12, 16, 17] and the use of antiplatelet agents [18–21] on all-cause or pneumonia-related mortality in this population have been inconsistent. Moreover, most prospective studies failed to analyze the time-dependent effect of the critical care management in patients admitted to ICU with pneumonia [12–14], which could result in misleading findings [22].

In this study, we evaluated the incidence of, and baseline and time-dependent risk factors for, mortality in patients admitted to ICU with pneumonia who participated in an international randomized controlled trial (RCT) of thromboprophylaxis, PROTECT (Prophylaxis for Thromboembolism in Critical Care Trial).

## Methods

### Patients and settings

This is a secondary analysis of PROTECT focusing on the patients admitted to ICU with a primary diagnosis of pneumonia, including hospital-acquired and community-acquired pneumonia. PROTECT was a multicenter RCT conducted in 67 ICUs in Canada, Australia, Brazil, Saudi Arabia, the United States and United Kingdom from 2006 to 2010, to investigate the efficacy of unfractionated heparin (UFH) versus dalteparin (a low–molecular weight heparin) for prevention of venous thromboembolism (VTE) (ClinicalTrials.gov Identifier: NCT00182143) [23, 24]. Inclusion criteria were age ≥ 18 years, weight ≥ 45 kg and expected ICU stay ≥ 3 days. Exclusion criteria were an admission diagnosis of trauma, orthopedic surgery, uncontrolled hypertension or neurosurgery, major hemorrhage within the previous week, pregnancy, stroke, thrombocytopenia, coagulopathy, and limitation of life-support. If patients needed anticoagulant therapy, had a contraindication to heparin or blood products, or were already enrolled in a related trial, then they were also excluded. Written informed consent was obtained from patients or surrogates prior to enrolment. Except for the blinded PROTECT study drug (i.e., UFH and dalteparin), all other aspects of care and management decisions including use of basic and advanced life support, antibiotics, fluid resuscitation and surgical interventions were at the discretion of the ICU team [24]. Research ethics board approval was obtained at each study center for the trial (details on the ethics approvals were presented in Additional file 1: Table S1). The Hamilton Integrated Research Ethics Board approved this secondary analysis and waived the need for additional consent.

### Outcome measures

Pneumonia was diagnosed if it represented the main reason for admission to the ICU as recorded by Research Coordinators who conferred with physicians [23, 24]. All patients were followed to hospital discharge to document vital status. The primary outcome was 90-day all-cause hospital mortality and the secondary outcome was 90-day all-cause ICU mortality [24]. For hospital mortality, patients who survived longer than 90 days in hospital or were discharged from hospital were censored; for ICU mortality, patients who survived longer than 90 days in ICU or were discharged from the ICU were censored.

### Independent variables

We collected data on both baseline characteristics and time-dependent variables in this study. Possible risk factors for mortality at baseline included sex, body mass index (BMI), APACHE (Acute Physiology and Chronic Health Evaluation) II score on admission, medical (versus surgical) admission, malignancy, end-stage renal disease, chronic hepatic failure, chronic respiratory failure, chronic heart failure, immunocompromised status, and personal or family history of VTE. We recorded daily interventions including use of inotropes or vasopressors, mechanical ventilation, dialysis, red blood cell transfusion, central venous catheterization, acetylsalicylic acid or clopidogrel, statins, stress ulcer prophylaxis, erythropoietin-stimulating agents, and activated protein C [23, 24].

### Statistical analysis

We presented frequency and percentages for categorical data, and mean and standard deviation (SD) or median and interquartile range (IQR) for continuous variables. Student’s *t*-test was used to compare continuous variables between the survivors and non-survivors, while chi-square test or Fisher’s exact test was used for categorical variables.

Survival curves for 90-day hospital mortality and ICU mortality were graphed using the Kaplan–Meier survivor function. Cox proportional hazards (PH) regression model was conducted to examine the relationship between independent variables and mortality, in which hazard ratios (HRs) were used to quantify the associations. Schoenfeld residuals were used to statistically test the PH assumption of Cox models [25]. We assessed the fixed effect of baseline variables including sex, BMI (per 5-point increase), APACHE II score (per 5-point increase), medical admission, malignancy, end-stage renal disease, chronic hepatic failure, chronic respiratory failure, chronic heart failure, immunocompromised status, and personal or family history of VTE. We treated the daily interventions as time-dependent covariates to investigate their time-dependent effect on hospital mortality and ICU mortality [22]. Univariate Cox regression models were initially conducted before choosing potentially significant risk factors with a *p*-value level < 0.20 for multivariable analyses. Variables with a two-sided *p*-value of < 0.05 in multivariable models were considered to be significant risk factors for mortality. The multicollinearity effect between risk factors in the model was detected using the variance inflation factor (VIF) of ≥ 4.

We performed a sensitivity analysis using data censored at 30 days for hospital and ICU mortality. All analyses were performed using STATA Version 12 (Stata Corp., College Station, TX, USA) with a significance level of 0.05.

## Results

There were 667 patients (43.8 % females) admitted to ICU with a primary diagnosis of pneumonia and included in this analysis. The mean age was 60.7 years (SD 16.0), BMI 28.2 (SD 7.7), and mean APACHE II score 21.3 (SD 7.5). Most patients (97.5 %) had a medical reason for ICU admission. The proportions of patients with baseline comorbidities including malignancy, end-stage renal disease, chronic hepatic failure, chronic heart failure and history of venous thromboembolism were not high; however, 17.5 % of patients had chronic respiratory disease and 14.7 % were immunocompromised (Table 1). Within the first 24 h of ICU admission, most patients received mechanical ventilation (93.1 %), central venous catheterization (79.8 %), and stress ulcer prophylaxis (88.4 %). There were 42.6 % of patients requiring inotropes or vasopressors, 25.3 % receiving acetylsalicylic acid or clopidogrel, and 21 patients (3.2 %) requiring dialysis within the first 24 h of admission (Table 1).

**Table 1:** Baseline characteristics and comparison between hospital survivors and non-survivors in patients admitted to ICU with pneumonia

Characteristics	Total patients (*n* = 667)^a^	Hospital non-survivors (*n* = 149)^b^	Hospital survivors (*n* = 518)^c^	*P*-value
Baseline variables				
Age (year): mean (SD)	60.7 (15.95)	68.4 (13.80)	58.5 (15.85)	<0.001^d^
Female: *n* (%)	292 (43.78)	54 (36.24)	238 (45.95)	0.035^e^
BMI (kg/m^2^): mean (SD)	28.2 (7.71)	27.7 (7.44)	28.3 (7.78)	0.36^d^
APACHE II score: mean (SD)	21.3 (7.53)	24.6 (7.36)	20.4 (7.32)	<0.001^d^
Medical admission: *n* (%)	650 (97.45)	147 (98.67)	503 (97.10)	0.29^e^
History of malignancy: *n* (%)	30 (4.50)	11 (7.38)	19 (3.67)	0.054^e^
End-stage renal disease: *n* (%)	10 (1.50)	5 (3.36)	5 (0.97)	0.034^f^
Chronic hepatic failure: *n* (%)	7 (1.05)	1 (0.67)	6 (1.16)	0.61^f^
Chronic respiratory disease: *n* (%)	117 (17.54)	39 (26.17)	78 (15.06)	0.002^e^
Chronic heart failure: *n* (%)	27 (4.05)	13 (8.72)	14 (2.70)	0.001^e^
Immunocompromise: *n* (%)	98 (14.69)	28 (18.79)	70 (13.51)	0.11^e^
Personal or family history of venous thromboembolism: *n* (%)	28 (4.20)	4 (2.68)	24 (4.63)	0.30^f^
Intervention within the first 24 h on admission: *n* (%)				
Inotropes/vasopressors	284 (42.58)	78 (52.35)	206 (39.77)	0.006^e^
Mechanical ventilation	621 (93.10)	140 (93.96)	481 (92.86)	0.64^e^
Dialysis	21 (3.15)	11 (7.38)	10 (1.93)	0.001^e^
Red blood cell transfusion	37 (5.55)	13 (8.72)	24 (4.63)	0.054^e^
Central venous catheterization	532 (79.76)	133 (89.26)	399 (77.03)	0.001^e^
Acetylsalicylic acid or clopidogrel	169 (25.34)	54 (36.24)	115 (22.20)	0.001^e^
Statins	104 (15.59)	28 (18.79)	76 (14.67)	0.22^e^
Stress ulcer prophylaxis	584 (88.35)	135 (91.84)	449 (87.35)	0.14^e^
Erythropoietin-stimulating agents	5 (0.76)	2 (1.35)	3 (0.59)	0.35^f^
Activated protein C	7 (1.05)	2 (1.35)	5 (0.97)	0.69^f^

*ICU* intensive care unit, *SD* standard deviation, *BMI* body mass index, *APACHE* Acute Physiology and Chronic Health Evaluation

^a^Median follow-up: 16 days, interquartile range: 10–29

^b^Median follow-up: 15 days, interquartile range: 8–26

^c^Median follow-up: 17 days, interquartile range: 10–30

^d^Student’s *t*-test

^e^Chi-square test

^f^Fisher’s exact test

**Fig. 1:**
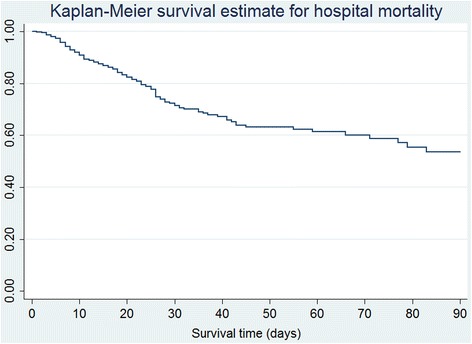
KM curve for 90-day Hospital mortality

Of the 667 patients during follow-up, 149 patients (22.3 %) died in hospital in total. The median hospital stay for survivors was 17 days (IQR: 10–30), and 15 days (IQR: 8–26) for non-survivors. Figure 1 displays the Kaplan-Meier survival curves for 90-day hospital mortality. Table 1 shows the unadjusted comparison between hospital survivors and non-survivors. At baseline, non-survivors were significantly older, were more likely to be male, and had higher APACHE II scores than survivors. More non-survivors had end-stage renal disease, chronic respiratory disease and heart failure. Within the first 24 h, hospital non-survivors were more likely to have received inotropes or vasopressors, dialysis, central venous catheterization, and acetylsalicylic acid or clopidogrel than survivors (*p*-values < 0.05) (Table 1).

Table 2 presents the results from univariate and multivariable analyses for 90-day hospital mortality. In the univariate analysis, higher APACHE II score, chronic heart failure, use of inotropes or vasopressors, dialysis, central venous catheterization, and acetylsalicylic acid or clopidogrel were found to be significantly related to increased risk of hospital mortality (*p*-values < 0.05). The multivariable analysis demonstrated significant risk factors for hospital mortality including male (HR = 1.5, 95 % CI: 1.1 – 2.2, *p*-value = 0.021), higher APACHE II score (HR = 1.2, 95 % CI: 1.1 - 1.4, *p*-value < 0.001 for per-5 point increase), chronic heart failure (HR = 2.9, 95 % CI: 1.6 - 5.4, *p*-value = 0.001), and dialysis (time-dependent effect: HR = 2.7, 95 % CI: 1.3 - 5.7, *p*-value = 0.008) (Table 2). No multicollinearity of independent variables or violation of the PH assumption in Cox models was observed.

**Table 2:** Results from univariate and multivariable analyses assessing the relationship between independent variables and 90-day hospital mortality

Independent variables	Hospital mortality^a^			
	Univariate analysis		Multivariable analysis^b^	
	HR (95 % CI)	*P*-value	HR (95 % CI)	*P*-value
Baseline variables				
Male	1.37 (0.97-1.92)	0.074	1.52 (1.07-2.17)	0.021
APACHE II score^c^	1.25 (1.13-1.38)	<0.001	1.23 (1.10-1.37)	<0.001
History of malignancy	1.84 (0.99-3.41)	0.053	1.61 (0.82-3.15)	0.16
End-stage renal disease	1.96 (0.79-4.88)	0.15	1.12 (0.38-3.25)	0.84
Chronic respiratory disease	1.40 (0.97-2.03)	0.073	1.36 (0.91-2.02)	0.14
Chronic heart failure	2.71 (1.53-4.79)	0.001	2.90 (1.57-5.37)	0.001
History of venous thromboembolism	0.50 (0.19-1.37)	0.18	0.64 (0.23-1.75)	0.38
Immunocompromise	1.40 (0.93-2.12)	0.12	1.33 (0.84-2.12)	0.23
BMI^c^	0.99 (0.89-1.10)	0.82	-^d^	-^d^
Medical admission	2.45 (0.61-9.90)	0.22	-^d^	-^d^
Chronic hepatic failure	0.65 (0.091-4.64)	0.67	-^d^	-^d^
Daily intervention variables				
Inotropes/vasopressors	1.59 (1.15-2.19)	0.005	1.24 (0.88-1.75	0.22
Dialysis	2.84 (1.52-5.30)	0.001	2.71 (1.30-5.65)	0.008
Red blood cell transfusion	1.64 (0.93-2.91)	0.090	1.22 (0.67-2.21)	0.51
Central venous catheterization	1.75 (1.04-2.94)	0.037	1.67 (0.99-2.80)	0.056
Acetylsalicylic acid or clopidogrel	1.44 (1.03-2.01)	0.034	1.28 (0.90-1.81)	0.17
Mechanical ventilation	1.34 (0.68-2.63)	0.40	-^d^	-^d^
Statins	1.27 (0.84-1.92)	0.26	-^d^	-^d^
Stress ulcer prophylaxis	1.43 (0.79-2.59)	0.23	-^d^	-^d^
Erythropoietin-stimulating agents	2.29 (0.57-9.30)	0.25	-^d^	-^d^
Activated protein C	1.64 (0.41-6.67)	0.49	-^d^	-^d^

*HR* hazard ratio, *CI* confidence interval, *BMI* body mass index, *APACHE* Acute Physiology and Chronic Health Evaluation

^a^There were 143 deaths for 90-day hospital mortality, ^b^No multicollinearity detected, ^c^Results for 5-point increase, ^d^Not included in the multivariable analysis

**Fig. 2:**
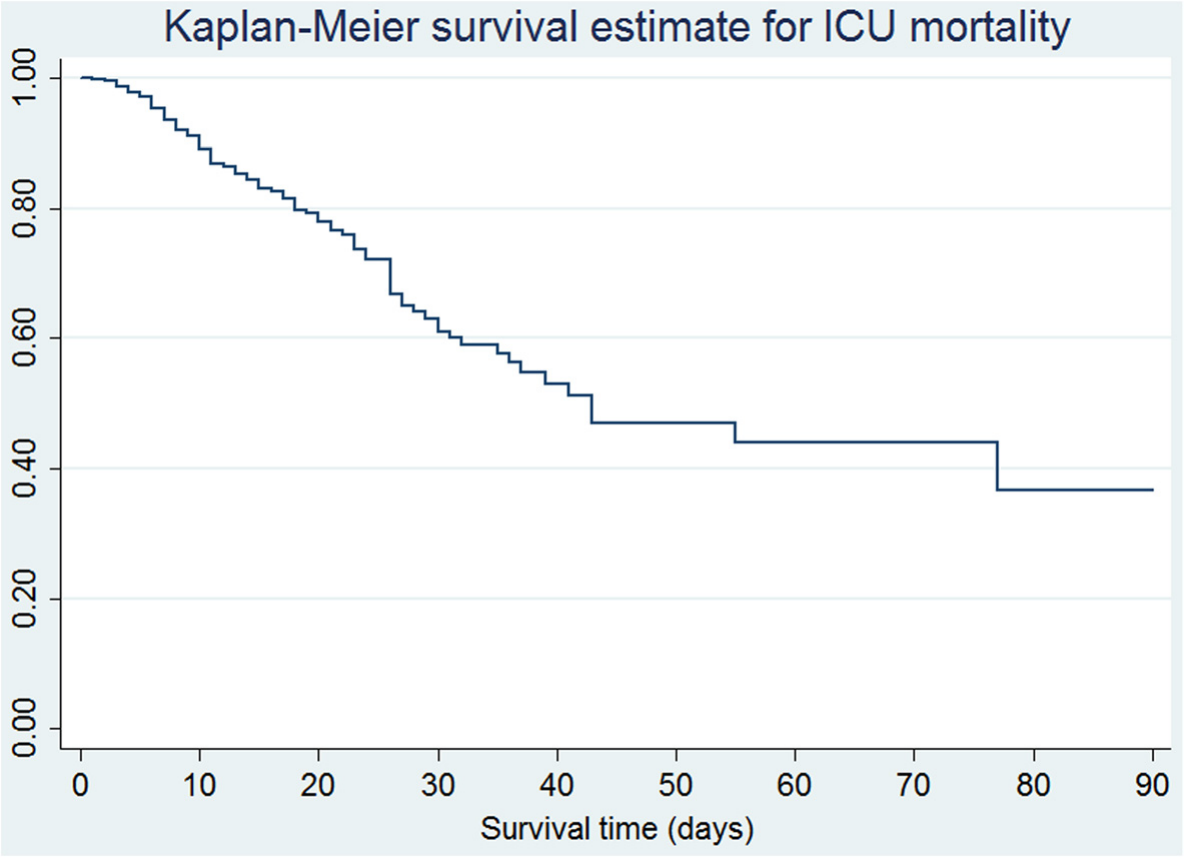
KM curve for 90-day ICU mortality

There were 111 deaths (16.6 %) during the ICU stay in this study, with a median survival time of 11 days (IQR: 6–24). Survivors stayed in ICU for a median of 8.5 days (IQR: 5–14). Fig. 2 shows the Kaplan-Meier survival curves for 90-day ICU mortality. Table 3 displays the results from Cox model evaluating the relationship between independent variables and 90-day ICU mortality. Male sex, APACHE II score, chronic heart failure and dialysis were found to be significantly associated with ICU mortality in the univariate analysis. In the multivariable analysis, only higher APACHE II score (HR = 1.2, 95 % CI: 1.1 - 1.4, *p*-value = 0.002 for per-5 point increase) and chronic heart failure (HR = 2.6, 95 % CI: 1.3 – 5.0, *p*-value = 0.004) were significantly associated with ICU mortality (Table 3).

**Table 3:** Results from univariate and multivariable analyses investigating the relationship between independent variables and 90-day ICU mortality

Independent variables	ICU mortality^a^			
	Univariate analysis		Multivariable analysis^b^	
	HR (95 % CI)	*P*-value	HR (95 % CI)	*P*-value
Baseline variables				
Male	1.52 (1.01-2.22)	0.042	1.41 (0.95-2.08)	0.096
APACHE II score^c^	1.20 (1.07-1.35)	0.002	1.22 (1.07-1.37)	0.002
Chronic respiratory disease	1.47 (0.97-2.23)	0.071	1.27 (0.81-1.97)	0.30
Chronic heart failure	2.48 (1.33-4.64)	0.004	2.58 (1.34-4.97)	0.004
History of venous thromboembolism	0.28 (0.068-1.16)	0.078	0.46 (0.11-1.91)	0.29
Immunocompromise	1.45 (0.91-2.33)	0.11	1.54 (0.94-2.52)	0.084
BMI^c^	0.97 (0.85-1.10)	0.61	-^d^	-^d^
History of malignancy	1.29 (0.57-2.94)	0.54	-^d^	-^d^
Medical admission	1.24 (0.31-5.04)	0.76	-^d^	-^d^
End-stage renal disease	1.49 (0.50-4.40)	0.47	-^d^	-^d^
Chronic hepatic failure	1.21 (0.17-8.67)	0.85	-^d^	-^d^
Daily intervention variables				
Inotropes/vasopressors	1.35 (0.92-1.98)	0.12	1.29 (0.88-1.92)	0.19
Dialysis	2.06 (1.04-4.09)	0.039	1.64 (0.77-3.52)	0.20
Red blood cell transfusion	1.60 (0.83-3.09)	0.16	1.30 (0.63-2.70)	0.48
Central venous catheterization	1.75 (0.93-3.27)	0.081	1.63 (0.92-2.87)	0.090
Erythropoietin-stimulating agents	2.66 (0.65-10.85)	0.17	1.15 (0.23-5.79)	0.86
Mechanical ventilation	1.05 (0.46-2.40)	0.91	-^d^	-^d^
Acetylsalicylic acid or clopidogrel	1.22 (0.82-1.81)	0.32	-^d^	-^d^
Statins	0.93 (0.56-1.55)	0.78	-^d^	-^d^
Stress ulcer prophylaxis	1.33 (0.64-2.74)	0.44	-^d^	-^d^
Activated protein C	0.86 (0.21-3.50)	0.84	-^d^	-^d^

*HR* hazard ratio, *CI* confidence interval, *BMI* body mass index, *APACHE* Acute Physiology and Chronic Health Evaluation

^a^There were 108 deaths for 90-day ICU mortality

^b^No multicollinearity detected

^c^Results for 5-point increase

^d^Not included in the multivariable analysis

Similar results from multivariable analyses were found in the sensitivity analysis when the data were censored at 30 days for hospital and ICU mortality (Table 4).

**Table 4:** Sensitivity analysis results from multivariable analyses evaluating the relationship between independent variables and 30-day hospital and ICU mortality

Independent variables	Hospital mortality^a^		ICU mortality^b^	
	HR (95 % CI)	*P*-value	HR (95 % CI)	*P*-value
Baseline variables				
Male	1.39 (0.96-1.96)	0.090	1.30 (0.85-2.04)	0.22
APACHE II score^c^	1.29 (1.14-1.47)	<0.001	1.20 (1.06-1.37)	0.004
History of malignancy	1.46 (0.70-3.03)	0.31	-^d^	-^d^
End-stage renal disease	1.13 (0.38-3.39)	0.82	-^d^	-^d^
Chronic respiratory disease	1.49 (0.98-2.27)	0.063	1.45 (0.92-2.28)	0.11
Chronic heart failure	2.25 (1.16-4.34)	0.017	2.57 (1.32-5.02)	0.006
History of venous thromboembolism	0.76 (0.28-2.09)	0.59	0.52 (0.13-2.12)	0.36
Immunocompromise	1.48 (0.91-2.41)	0.12	1.43 (0.85-2.39)	0.17
Daily intervention variables				
Inotropes/vasopressors	1.21 (0.84-1.75)	0.31	1.07 (0.71-1.62)	0.75
Dialysis	2.29 (1.03-5.11)	0.042	1.33 (0.59-2.98)	0.49
Red blood cell transfusion	1.20 (0.62-2.30)	0.59	1.38 (0.67-2.84)	0.39
Central venous catheterization	1.50 (0.86-2.63)	0.15	1.12 (0.62-2.01)	0.72
Acetylsalicylic acid or clopidogrel	1.36 (0.93-2.00)	0.11	-^d^	-^d^
Erythropoietin-stimulating agents	-^e^	-^e^	1.39 c0.32-6.04)	0.66

*HR* hazard ratio, *CI* confidence interval, *BMI* body mass index, *APACHE* Acute Physiology and Chronic Health Evaluation

^a^There were 122 deaths for 30-day hospital mortality; No multicollinearity detected

^b^There were 97 deaths for 30-day hospital mortality; No multicollinearity detected

^c^Results for 5-point increase

^d^Not included in the multivariable analysis for ICU mortality

^e^Not included in the multivariable analysis for hospital mortality

## Discussion

In this multicenter trial database analyzed to assess risk factors for mortality in critically ill patients admitted to ICU with pneumonia, we found that 17 % of patients died in ICU and 22 % died in the hospital. Higher APACHE II score on admission to the ICU and chronic heart failure were significantly related to risk of death in the ICU. Higher APACHE II score, male sex, chronic heart failure, and dialysis were independently associated with risk of death in hospital.

The substantial mortality rate of patients admitted to ICU with pneumonia has been described in prior studies, despite heterogeneous healthcare systems, study designs, treatments, and guideline adherence [8, 26–28]. A large multicenter international cohort study of 1166 patients admitted to ICU with pneumonia in Europe reported an ICU mortality of 19 % and a hospital mortality of 24 % [8], which is comparable to our study. In our study, males were more likely to die in hospital than females (HR = 1.5). The impact of gender on mortality in critically ill patients with pneumonia remains controversial. Some studies reported a lower risk of death in females [6, 29, 30], whereas others demonstrated a higher mortality rate for women [16, 31]. It has been postulated that gender differences in mortality may relate to sex steroids [16, 32]. Female sex hormones appear to be immune-stimulatory, while male sex hormones appear to be immunosuppressive, potentially partially explaining the lower risk of mortality in critically ill female patients [30, 33]. However, the result should be interpreted with caution because our analyses could not be adjusted for other potential confounding factors including smoking and alcohol abuse that might influence the effect of sex on mortality.

Higher APACHE II score on admission to the ICU and chronic heart failure have previously been found to be independently associated with increased risk of death [7, 8, 34, 35]. A large population-based cohort study in Denmark reported a 30-day mortality risk ratio of 1.4 in heart-failure patients hospitalized for pneumonia compared to patients without pneumonia [35], which was consistent with our findings that baseline chronic heart failure predicted worse survival in ICU and hospital (Table 2, Table 3). Similarly, the need for dialysis was associated with an increased risk of death in patients with pneumonia, as has been shown for critically ill patients in general [7, 13, 36].

In contrast to other previous reports [8, 34, 37], we found no significant relationship between mechanical ventilation and risk of death in this study (Tables 2 and 3). This may be because almost all of the patients received mechanical ventilation (93 %, Table 1). It is unlikely that a variable will be a significant risk factor for an outcome in regression analysis if the variable characterizes a very high proportion in the population [38]. Use of antiplatelet agents or statins was not related with hospital or ICU mortality in this study. Prior studies have shown that antiplatelets and statins could modulate inflammatory reactions to attentuate the severity of pneumonia and the development of organ failure in critically ill patients, which was therefore associated with improved outcomes [19–21]. Some observational studies reported a significant inverse relationship between mortality and antiplatelet agents [20, 21, 39] or statins [19, 40]; however, our findings agree with the results from other randomized trials [18, 41, 42] that did not support the beneficial effect on short-term all-cause or pneumonia-related mortality. More evidence is needed to further clarify the possible mechanisms of action and possible clinical effects of antiplatelet agents and statins on survival during critical illness in ICU patients with pneumonia.

Strengths of this study include the multicenter design and heterogeneous group of patients from six countries. Associations between risk of mortality in patients admitted to ICU with pneumonia and gender and use of antiplatelet agents were evaluated, given the inconsistent findings from other studies. In this study we focused on patients admitted to ICU with pneumonia, because previous studies typically investigated how to prevent pneumonia after ICU admission, or how to improve survival for all the hospitalized patients with pneumonia (rather than those critically ill patients requiring ICU admission). We used rigorous analytic approaches to assess the relationship between baseline and time-dependent risk factors and mortality to avoid potential confounding and misleading findings. Furthermore, our study yielded similar results to the 90-day mortality in the 30-day mortality sensitivity analysis, which may provide some value to the existing literature because studies of ICU patients with infection have typically focused on the shorter term follow-up [11, 18, 41, 42]. There are several limitations of our study. First, because the diagnosis of pneumonia was made by physicians’ in practice, we lack specific diagnostic criteria used in each case (e.g., type of chest x-ray infiltrate). We also acknowledge that pneumonia may be over-diagnosed in patients admitted to ICU [43, 44]; however, disincentivized reporting of pneumonia as a quality metric in many settings indicates that pneumonia may also be under-diagnosed and under-reported as well. Furthermore, although both hospital-acquired pneumonia and community-acquired pneumonia were included in this analysis, we could not distinguish them, as the diagnosis was not centrally adjudicated. Therefore we could not evaluate whether their risk factors for mortality differed. Similarly due to no central adjudication of infection, some patients may have had underlying pneumonia but received a primary diagnosis of septic shock or acute respiratory distress syndrome on ICU admission, and were not categorized as having pneumonia in this study. Moreover, no data on microbiology, degree of hypoxia, respiratory mechanics, inflammatory biomarkers, or antimicrobials were documented in this thromboprophylaxis trial; treatment was at the physicians’ discretion, replicating practice [24]. Because there is no well-validated classification for cause-specific mortality for critically ill patients, we used all-cause mortality, as per the trial database [23]. Using data from a randomized thromboprophylaxis trial with topic-specific eligibility criteria [23, 24] could limit the generalizability of these findings. The number of deaths (143 for 90-day hospital mortality, Table 2; 108 for 90-day ICU morality, Table 3) resulted in imprecise estimates of the strength of the risk factors in our multivariable analyses, rendering the regression analysis potentially underpowered to assess these or other risk factors.

## Conclusion

In summary, in this study using data from a multicenter thromboprophylaxis trial, we found that male sex, higher APACHE II score on admission, chronic heart failure, and dialysis were independently associated with risk of hospital mortality in patients admitted to ICU with pneumonia. None of these are modifiable risk factors. Findings from this study may help to inform clinicians about prognostic factors for patients with pneumonia and identify higher-risk patients who may benefit from closer monitoring. While high illness severity score, presence of a serious comorbidity (heart failure) and need for an advanced life support (dialysis) are not unexpected risk factors of mortality, male sex might necessitate further exploration. More studies are warranted to clarify the effect of these risk factors on survival in critically ill patients admitted to ICU with pneumonia.

## Abbreviations

APACHE, acute physiology and chronic health evaluation; BMI, body mass index; CI, confidence interval; HR, hazard ratio; IQR, interquartile range; ICU, intensive care unit; PH, proportional hazards; PROTECT, Prophylaxis for thromboembolism in critical care trial; RCT, randomized controlled trial; SD, standard deviation; UFH, unfractionated heparin; VIF, variance inflation factor

## Additional file

Additional file 1: Table S1. Details of PROTECT Research Ethics Board (REB) approvals. (DOCX 19 kb)
