# Erratum to: Risk factors for mortality in patients admitted to intensive care units with pneumonia

**DOI:** 10.1186/s12931-016-0444-2

**Published:** 2016-10-07

**Authors:** Guowei Li, Deborah J. Cook, Lehana Thabane, Jan O. Friedrich, Tim M. Crozier, John Muscedere, John Granton, Sangeeta Mehta, Steven C. Reynolds, Renato D. Lopes, Francois Lauzier, Andreas P. Freitag, Mitchell A. H. Levine, the PROTECT Investigators for the Canadian Critical Care Trials Group, the Australian and New Zealand Intensive Care Society Clinical Trials Group

**Affiliations:** 1 Department of Clinical Epidemiology & Biostatistics, McMaster University, 501-25 Charlton Avenue East, Hamilton, ON L8N 1Y2 Canada; 2 St. Joseph’s Healthcare Hamilton, McMaster University, 501-25 Charlton Avenue East, Hamilton, ON L8N 1Y2 Canada; 3 Department of Medicine, McMaster University, Hamilton, ON Canada; 4 Interdepartmental Division of Critical Care, Hamilton Health Sciences, Hamilton, ON Canada; 5 Interdepartmental Division of Critical Care Medicine, University of Toronto, Toronto, ON Canada; 6 St. Michael’s Hospital, University of Toronto, Toronto, ON Canada; 7 Intensive Care Unit, Monash Medical Centre, Melbourne, VIC Australia; 8 Department of Critical Care Medicine, Queens University Kingston, Kingston, ON Canada; 9 University Health Network, University of Toronto, Toronto, ON Canada; 10 Mount Sinai Hospital, University of Toronto, Toronto, ON Canada; 11 Division of Critical Care, Department of Medicine, University of British Columbia, Vancouver, BC Canada; 12 Duke Clinical Research Institute, Duke University, Durham, NC USA; 13 Department of Anesthesiology and Critical Care Medicine, Division of Critical Care Medicine, Université Laval, Québec, Canada; 14 Department of Clinical Epidemiology & Biostatistics, McMaster University, 25 Main St. West, Suite 2000, 20th floor, Hamilton, ON L8P 1H1 Canada; 15 Centre for Evaluation of Medicines, St. Joseph’s Healthcare Hamilton, 25 Main St. West, Suite 2000, 20th floor, Hamilton, ON L8P 1H1 Canada

## Erratum

In the original publication of this article [1], one of the co-authors’ names was listed incorrectly. ‘Lauzier Francois’ should have been listed as ‘Francois Lauzier’.
