# Supplementary material for: Risk factors for mortality in patients admitted to intensive care units with pneumonia
Source: Respir Res. 2016 Jul 11;17:80. doi: 10.1186/s12931-016-0397-5 (PMC4940754; doi:10.1186/s12931-016-0397-5)
Supplement: Additional file 1: Table S1. — Details of PROTECT Research Ethics Board (REB) approvals. (DOCX 19 kb) [file 12931_2016_397_MOESM1_ESM.docx]

**Supplementary Table 1.** Details of PROTECT Research Ethics Board (REB) approvals

| **Institution** | **Name of REB** | **REB Number** |
| --- | --- | --- |
| St Joseph’s Hospital | St Joseph’s Healthcare Hamilton REB | 05-2572 |
| QEII, Halifax | Capital Health Research Ethics Board | 2005-321 |
| Hamilton Health Science-Hamilton General Hospital | Hamilton Health Science Research Ethics Board | 05-429 |
| Hamilton Health Science-McMaster  Medical Center | Hamilton Health Science Research Ethics Board | 05-429 |
| University Health Network-Toronto General Hospital | University Health Network Research Ethics Board | 05-0934-A |
| Centre hospitalier affi llie universitaire de Quebec-Enfant Jesus Hospital | Centre hospitalier affi llie universitaire  de Quebec-Research Ethics Board | PEJ-338 |
| Hopital Charles LeMoyne | Comite d’ethique de la recherché Hopital  Charles LeMoyne | 2005-06-52 |
| Ottawa Hospital-Civic Campus | The Ottawa Hospital Research Ethics Board | 2005872-01H |
| Ottawa Hospital-General Campus | The Ottawa Hospital Research Ethics Board | 2005872-01H |
| Hopital Maisonneuve Rosemont | Comite d’ethique de la recherché de l’hopital Maisonneuve Rosemont | #05093 |
| Hopital du Sacre Coeur de Montreal | Comite d’ethique de la recherché Sacre Couer Hospital | C.E 2005-12-80 |
| Mount Sinai Hospital | Mount Sinai Hospital Research Ethics Board | 05-0259-A |
| Sunnybrook Health Science Center | Sunnybrook Health Science Center Research  Ethics Board | 043-2006 |
| St Michael’s Hospital | S t Michael’s Hospital Research Ethics Board | 06-007 |
| Kingston General Hospital | Queens University Health Sciences and Affiliated Teaching Hospitals Research Ethics Board | DMED-915-05 |
| Hospital Moinhos de Vento | Comite de Etica em Pesquisa e Comissao Cientifi ca Hospital Moinhos de Vento | CEP-IEP HMV:2006/37 |
| Hospital ProCardiaco | Comite de Etica em Pesquisa e Comissao Cientifi ca Hospital ProCardiaco | CEP 236 |
| Royal Columbian Hospital | Fraser Health Research Ethics Board | FHREB 2005-96 |
| Vancouver General Hospital | The University of British Columbia Clinical Research  Ethics Board | H05-70623 |
| St Paul’s Hospital | The University of British Columbia Providence Health Care Research Ethics Board | H05-50274 |
| Foothills Hospital | Conjoint Health Ethics Research Board | E-20147 |
| Royal Alexandra Hospital | The Health Research Ethics Board | Pro00001957 |
| University Health Network-Toronto Western Hospital | University Health Network Research Ethics Board | 05-0934-A |
| The Alfred Hospital | The Alfred Ethics Committee | 236/05 |
| Royal Melbourne Hospital | Research Directorate-Human Ethics Committee, Melbourne Health | 2005.224 |
| MD Anderson Cancer Center | MD Anderson Cancer Center Institutional Review Board | 2008-0466 |
| Irmandade da Santa Casa de Misericordia de Porto Alegre | Comite de Etica em Pesquisa e Comissao Cientifi ca Irmandade da Santa Casa de Misericordia de Porto Alegre | 1368/06 |
| Hospital do Coracao | Comite de Etica em Pesquisa e Comissao Cientifi ca Hospital do Coracao | 036/2007 |
| St John’s Mercy Medical Center | St John’s Mercy Medical Center Institutional  Review Board | 09-021 |
| L’Hopital Laval | Comite d’ethique de la recherché de l’hopital Laval | 20183 |
| Hamilton Health Science-Henderson Hospital | Hamilton Health Science Research Ethics Board | 05-429 |
| Hospital Sao Paulo | Comite de Etica em Pesquisa Hospital Sao Paulo | CEP 1523/06 |
| University of Alberta Medical Research Center | Health Research Ethics Board | 6174 |
| Mayo Clinic | Mayo Clinic Institutional Review Board | 08-002579 |
| Austin Health | Austin Health Human Research Ethics Committee | H2006/02436 |
| Boxhill Hospital | Eastern Health Research and Ethics Committee | E70/0506 |
| Frankston Hospital | Peninsula Health Research and Ethics Committee | 2005-59 |
| Monash Medical Center | Southern Health Research Ethics Committee | 05173B |
| Nepean Hospital | Human Research Ethics Committee,  Nepean Campus | 06/030 |
| Royal Adelaide Hospital | Royal Adelaide Hospital Research  Ethics Committee | 070323 |
| King Faisal and Specialist Research Center | King Faisal and Specialist Research Center –Institutional Review Board | 2006-02 |
| Royal Prince Alfred Hospital | Sydney South West Area Health Service Ethics Review Committee | X06-0047 |
| Peter Lougheed Hospital | Conjoint Health Ethics Research Board | E-20147 |
| Bendigo Health Center | Bendigo Health Human Research Ethics Committee | 1/2006 |
| Blacktown Hospital | Human Research Ethics Committee, Nepean Campus | 06/030 |
| Flinders Medical Center | Flinders Clinical Research Ethics Committee | 120/056 |
| Geelong Hospital | Barwon Health Research and Ethics  Advisory Committee | 06/04 |
| McGill University Health Center-Montreal General | Center Universitaire de Sante McGill Bureau d’ethique de la Recherche | BMB 07-00 |
| Riyadh Military Hospital | Riyadh Military Hospital Research and Ethical Committee | 338-2008 |
| Dandenong Hospital | Southern Health - Human Research Ethics Committee B | 05173B |
| King Fahad Medical City | Institutional review Board King Fahad Medical City | 004-08 |
| University of Wollongong | University of Wollongong South Eastern Sydney Illawarra NSW Health Human Research Ethics Committee | 236/05 |
| Vancouver Island Health Authority | Research Review and Ethical Approval Committee | 2006-21 |
| Guy’s and St Thomas Hospital | Guy’s and St Thomas Hospital National Foundation Trust Research and Development | 09/H0802/022 |
| Center Hospitalier Universitaire de Sherbrook | Comite d’ethique de la recherché en Santechez l’Human | 07-019 |
| McGill University Health Center-Royal Victoria Hospital | Center Universitaire de Sante McGill Bureau  d’ethique de la Recherche | BMB 07-003 |
| Guelph General Hospital | Guelph General Hospital Research Ethics Board | 7-010 |
| Grand River Hospital | Tri-Hospital Research Ethics Board | 07-150 |
| St Boniface Hospital | Bannatyne Campus Research Ethics Board | B2007:085 |
| Lakeridge Health | Lakeridge Health Research Ethics Board | 2008-007 |
| Rhode Island Hospital | Lifespan Institutional Review Board | 00004624 |
| Lyell McEwin Hospital | Southern Health Research Ethics Committee | 05173B |
| Surrey Memorial Hospital | Fraser Health Research Ethics Board | FHREB 2005-96 |
| King Abdulaziz Medical City Hospital | King Abdulaziz Medical City Hospital Institutional Review Board | RC07/015 |
| London Health Science Center-University Campus | University of Western Ontario Research Ethics Board | 16565 |
| King Abdulaziz University Hospital | King Abdulaziz University Hospital Bioethical and Research Committee | 225-08 |
